# Comparison of pregnancy outcomes between people with perinatally acquired HIV, horizontally acquired HIV and those without HIV

**DOI:** 10.1097/QAD.0000000000004114

**Published:** 2025-03-13

**Authors:** Shivani Shah, Charlotte-Eve Short, Graham Taylor, Hermione Lyall, Caroline Foster

**Affiliations:** a Imperial College Healthcare NHS Trust; b Imperial College London; c Chelsea and Westminster NHS Foundation Trust, London, UK.

## Abstract

A retrospective case-controlled study compared pregnancy outcomes between people with perinatally acquired HIV (PaHIV), horizontally acquired HIV (HaHIV), and those without HIV. PaHIV were more likely to be viraemic in early pregnancy than HaHIV. When matched for age and ethnicity, babies born to PaHIV were more likely to be premature, small for gestational age, delivered by caesarean section and require enhanced neonatal and social care involvement than infants born to age/ethnically matched HIV-uninfected individuals.

Pregnancies in people with HIV in England have declined from 1400 in 2009 to 607 in 2020; however, people born with perinatally acquired HIV (PaHIV) represent an increasing proportion; from 1.5% in 2015 to 5.6% in 2020 [1]. Age at pregnancy and ethnicity affect pregnancy outcomes in the general population; pregnant PaHIV are younger than those with horizontally acquired HIV (HaHIV) and are more likely to be of black ethnicity, a group who, in the general UK population, experience double the rate of infant mortality; 6.4/1000 live births vs. 3.7/1000 for all UK ethnicities [2].

Despite suppressive antiretroviral therapy (ART), pregnant people with HIV (PHIV) have an increased risk of premature, low birth weight (LBW), small for gestational age (SGA) infants and adverse psychosocial outcomes [3–8]. Early data suggests route of HIV acquisition impacts perinatal outcomes with PaHIV people having been exposed to more ART regimens and more likely to have viraemia, immunosuppression and HIV associated drug resistance mutations than those with HaHIV; although obstetric outcomes appear broadly similar when adjusted for age and ART [7,9–12].

In this single centre study in London, UK, we compared maternal and infant outcomes in pregnant people with HIV by route of acquisition (PaHIV vs. HaHIV matched 1 : 1) and with an HIV-uninfected group (PaHIV vs. HIV-uninfected) matched 1 : 2 by age and ethnicity. All PaHIV pregnancies between 2015 and 2022 were included and matched by nearest date of delivery to HaHIV and HIV-uninfected controls. Data on demographics, immune and virologic parameters, and obstetric and infant outcomes extracted from electronic health records are documented in Table 1. Data were analysed in SPSS version 28 for MacIntosh and were compared between two groups using the Mann–Whitney *U* test and across three groups with the Kruskal–Wallis test. Categorical data outcomes reported as proportions were compared between two groups by the Fisher Exact test and across three groups by the chi-squared test. Binary outcomes within and between group categories were compared by odds ratios (Table 1).

**Table 1:** Comparison of the demographics, antenatal, perinatal and postnatal outcomes between people with perinatally acquired HIV, horizontally acquired HIV and those without HIV.

	PaHIV (*n* = 19)	HaHIV (*n* = 17)	HIV− (*n* = 33)	*P* value^∗^	PaHIV vs. HaHIV [OR (95% CI)]	PaHIV vs. HIV− [OR (95% CI)]
Aage (years) at registering for antenatal care median (IQR)	25 (4)	35 (9)	24 (4)	<0.001		
Maternal BMI (kg/m^2^)						
<18	0	1 (6%)	1 (3%)	0.075		
18–24	15 (79%)	6 (35%)	15 (46%)			
25–29	1 (5%)	5 (29%)	11 (33%)			
>30	3 (16%)	5 (29%)	6 (18%)			
Ethnicity						
Black	17 (90%)	16 (94%)	33	0.51		
South Asian	0	1 (6%)	0			
Missing data	2 (10%)	0	0			
Maternal learning difficulties	2 (10.5%)	1 (6%)	0	0.14		
Maternal alcohol, smoking, drug use	4 (21%)	2 (12%)	2 (6%)	0.20		
Missing data	2 (10%)	0	0			
Gestation at registering for antenatal care maternity booking						
<13 weeks	11 (65%)	8 (47%)	21 (64%)	0.66		
13–26 weeks	6 (35%)	8 (47%)	9 (27%)			
>27 weeks	0	1 (6%)	3 (9%)			
Maternal hypertension	3 (16%)	1 (6%)	1 (3%)	0.17	3.4 (0.3–36.8)	
					*P* = 0.6	
CD4^+^ cell count at time of maternity booking (cells/μl)						
CD4^+^ >200	14 (74%)	17 (100%)	–	0.10		
CD4^+^ <200	4 (21%)	0	–			
Missing data	1 (5%)	0	–			
Detectable viral load on registering for antenatal careat booking (>50 copies/ml)	6 (31.6%)	3 (17.6%)	–	0.005	2.7 (0.6–12.9)	
					*P* = 0.27	
Detectable viral load at delivery (>50 copies/ml)	0	1 (5.9%)	–	1.0	1.8 (0.15–21)	
					*P* = 1.00	
ART by anchor class with two NRTI^∗^						
Boosted PI^∗^	8 (37%)	7 (38%)	–	0.61		
INSTI^∗^	6 (32%)	1 (6%)	–	0.09		
NNRTI^∗^	1 (5%)	3 (25%)	–	0.33		
Boosted PI + INSTI^∗^	3 (21%)	1 (6%)	–	0.61		
LA-ART	1 (5%)		–	1.00		
Triple NRTI		3 (19%)	–	0.09		
+ Entry inhibitor		1 (6%)	–	0.47		
Cumulative drug resistance mutations by drug class						
NNRTI alone	4 (21%)	0	–			
PI alone	1 (5%)	0	–			
NNRTI + NRTI	5 (27%)	0	–	0.02		
NNRTI + PI	1 (5%)	0	–			
Wild type	8 (42%)	16 (94%)	–			
Not available	0	1 (6%)	–			
Mode of delivery						
Elective C/S	6 (32%)	7 (41%)	0	<0.001		
Emergency C/S	5 (26%)	3 (18%)	5 (15%)	0.61	1.7 (0.3–8.4)	2.0 (0.5–8.1)
					*P* = 0.7	*P* = 0.47
Vaginal delivery	8 (42%)	7 (41%)	28 (85%)	<0.001	1.04 (0.3–3.9)	0.13 (0.04–0.5)
					*P* = 1.0	*P* = 0.002
Preterm birth	6 (32%)	3 (20%)	3 (10%)	0.11	2.3 (0.5–11.4)	5.0 (1.1–23.3)
					*P* = 0.44	*P* = 0.05
Small for gestational age (SGA)						
Yes	5 (26%)	0	5 (15%)	0.037	∼	2.8 (0.7–11.8)
No	10 (53%)	17	28 (85%)			*P* = 0.25
Missing data	4 (21%)	0	0			
Low birth weight (LBW)	8 (47%)	4 (23%)	4 (12%)	0.03	3.6 (0.7–17.8)	5.6 (1.3–23.8)
					*P* = 0.14	*P* = 0.03
NICU admission	5 (29%)	2 (12%)	2 (6%)	0.02	8.8 (0.8–78.8)	7.8 (1.3–46.3)
					*P* = 0.08	*P* = 0.02
Any breast/chest feeding	0	5 (31%)	32 (97%)	<0.001	∼	
Child protection plan	5 (26%)	1 (6%)	1 (3%)	0.02	5.7 (0.6–55)	11.4 (1.2–107)
					*P* = 0.51	*P* = 0.02

∼ indicates incalculable OR as observed outcome data = 0. ART, antiretroviral therapy; C/S, caesarean section; HaHIV, horizontally acquired HIV; HIV−, without HIV; INSTI, integrase strand transfer inhibitor; IQR, interquartile range; IUD, intrauterine death; LA-ART, long-acting antiretroviral treatment; LBW, weight at birth <2.5 kg); NICU, neonatal ICU; NNRTI, nonnucleoside reverse transcriptase inhibitor; NRTI, nucleoside reverse transcriptase inhibitor; PaHIV, perinatally acquired HIV; PI, protease Inhibitor; SGA, birthweight less than 10th percentile for gestational age.

∗ *P* value for comparison across 3 groups (PaHIV, HaHIV and HIV−) using Kruskal–Wallis test for interval data and chi-squared test for categorical data.

All people were of black ethnicity apart from one, with HaHIV being 10 years older than PaHIV and age-matched HIV-uninfected individuals (24 vs. 34 years *P* < 0.001). PaHIV were more likely to have social care involvement (PaHIV vs. HaHIV and HIV-uninfected, *P* = 0.05) and infants subject to a child protection plan (PaHIV vs. HaHIV and HIV-uninfected, *P* = 0.02). Twice the number of PaHIV had detectable viraemia at the time of registering for antenatal care (31.6 vs. 17.6% HaHIV, *P* = 0.005) and those with viraemia were three times more likely to have severe immunosuppression (CD4^+^ cell count <200 cells/μl) compared with HaHIV with viraemia [OR 3.0 (95% CI 1.0–9.3), *P* = 0.005]. Viraemia was associated with documented poor adherence to ART in all cases. No women with HaHIV had documented HIV-1-associated drug resistance mutations (DRM) in contrast to 11 of 19 (58%) with PaHIV having any DRM with 6 of 19 (32%) having dual class resistance most frequently nucleoside reverse transcriptase inhibitor (NRTI) and nonnucleoside reverse transcriptase inhibitor (NNRTI) (5/19; 27%). All bar one individual (HaHIV) achieved viral suppression by delivery with no infant infections. There was no significant difference in ART by anchor class (PaHIV vs. HaHIV), although there was a trend towards increased use of integrase inhibitors (INSTI) (32% PaHIV, 6% HaHIV) and complex ART (boosted protease inhibitor + INSTI) in PaHIV (21 vs. 6% HaHIV). People with HIV were more likely to deliver by caesarean section, emergency or elective, compared to HIV-uninfected (57.5 vs. 15.2%; *P* < 0.001) although there was no difference in rates of emergency caesarean section between the three cohorts (*P* = 0.61).

PaHIV were significantly more likely to deliver LBW infants compared to age and ethnically matched HIV-uninfected young people (*P* = 0.03), with a trend towards increased preterm/LBW infants for PaHIV compared to HaHIV. PaHIV were significantly more likely to have SGA infants when compared to HaHIV and HIV-uninfected controls (*P* = 0.037). 29.4% of PaHIV infants required neonatal intensive care (NICU) compared to 11.7% HaHIV and 6% HIV-uninfected (*P* = 0.02). One intrauterine death occurred at 28 weeks’ gestation following placental abruption to a PaHIV with a viral load more than 1 million HIV RNA copies/ml plasma and CD4^+^ cell count less than 200 cells/μl at the time of registering for antenatal care but undetectable viral load at delivery.

People with HIV were less likely to breast/chestfeed their infants (*P* < 0.001), and whilst breast/chestfeeding is not recommended for people with HIV in the UK, since 2011, those on suppressive ART who choose to breast/chest feed are supported to do so [13].

In this study, PaHIV were more likely to have complex neonatal outcomes, deliver premature, LBW and SGA infants, with higher rates of both neonatal and social care involvement when compared to HIV-uninfected controls matched for both age and ethnicity, and adds weight to previous studies [3–5]. PaHIV were younger, were more likely to have detectable viral load, immunosuppression and archived DRMs than HaHIV, but reassuringly, all PaHIV achieved viral suppression by delivery, with no vertical transmissions. However, this required high levels of multidisciplinary input, including social care, and highlights the need for early engagement and enhanced support for PaHIV even as compared to HaHIV. There was a trend to more adverse infant outcomes in PaHIV compared to HaHIV and previous studies have found that compared to HaHIV, PaHIV was a risk factor for SGA, with maternal viral suppression being a protective factor for infant growth [14]. SGA at birth has long-term implications into adulthood, including increased risk of cardiovascular disease, diabetes, obesity, and lower cognitive performance, even when controlled for socioeconomic status.

High-income settings have extremely small numbers of infants born to PaHIV, and the real burden of adverse neonatal outcomes in this population will be seen in sub-Saharan Africa. Sub-Saharan Africa has the highest neonatal and child mortality rates globally, and a systematic review of almost 400,000 deliveries demonstrated an association between HIV and ART contributing to adverse perinatal outcomes associated with neonatal mortality [6]. As the global population of adults with PaHIV grows, it is important to monitor outcomes for third-generation infants born in high-prevalence settings and provide appropriate antenatal and postnatal support.

Our study is limited by the small, single-centre sample size and retrospective study design. Potential confounding factors, including maternal mental health, social support, socioeconomic status and comorbidities, were not addressed. This highlights the pressing need for larger cohort studies, and the importance of data disaggregated by maternal route of HIV acquisition, as increasing numbers of young people growing up with HIV enter adult services.

## Acknowledgements

We would like to thank Nicola Sale for providing the data for the HIV uninfected cohort.

Subject consent: no consent was required.

Ethics: ethical approval was not required as per UK Health Research Authority guidance for use of anonymized, routinely collected clinical data.

### Conflicts of interest

There are no conflicts of interest.
